# Safety of endovascular therapy in ischemic stroke patients ⩾90 years: A cohort study from the EVA-TRISP collaboration

**DOI:** 10.1093/esj/23969873251360607

**Published:** 2026-01-01

**Authors:** Jasmine Jost, Lukas Enz, Martina B Goeldlin, Philipp Baumgartner, Davide Strambo, Nabila Wali, Nicolas Martinez-Majander, Georg Kägi, Laura Vandelli, Christoph Riegler, Danna Krupka, Matteo Paolucci, Mauro Magoni, Giovanni Bianco, Hamza Jubran, Dejana R Jovanovic, Tomas Klail, Laura P Westphal, Alexander Salerno, Leon A Rinkel, Laura Mannismäki, Tolga Dittrich, Livio Picchetto, Regina von Rennenberg, Miguel Serôdio, Stefano Forlivesi, Dikran Mardighian, Carlo W Cereda, Ronen R Leker, Visnja Padjen, Mira Katan, Marios-Nikos Psychogios, Urs Fischer, Tomas Dobrocky, Mirjam R Heldner, Patrik Michel, Paul J Nederkoorn, Sami Curtze, Gian Marco De Marchis, Guido Bigliardi, Christian H Nolte, João Pedro Marto, Andrea Zini, Alessandro Pezzini, Susanne Wegener, Marcel Arnold, Stefan T Engelter, Henrik Gensicke

**Affiliations:** Stroke Center and Department of Neurology, University Hospital Basel and University of Basel, Basel, Switzerland; Neurology and Neurorehabilitation, University Department of Geriatric Medicine FELIX PLATTER, University of Basel, Basel, Switzerland; Stroke Center and Department of Neurology, University Hospital Basel and University of Basel, Basel, Switzerland; Department of Neurology and University Institute of Diagnostic and Interventional Neuroradiology, Inselspital, Bern University Hospital and University of Bern, Bern, Switzerland; Department of Neurology, University Hospital Zurich and University of Zurich, Zurich, Switzerland; Stroke Center, Neurology Service, Lausanne University Hospital and University of Lausanne, Lausanne, Switzerland; Department of Neurology, Amsterdam UMC Location University of Amsterdam, Amsterdam, The Netherlands; Neurology, University of Helsinki and Helsinki University Hospital, Helsinki, Finland; Department of Neurology and University Institute of Diagnostic and Interventional Neuroradiology, Inselspital, Bern University Hospital and University of Bern, Bern, Switzerland; Department of Neurology and Stroke Center, University Teaching and Research Hospital, Kantonsspital St. Gallen, St. Gallen, Switzerland; Neurology - Stroke Unit, Department of Neuroscience, Ospedale Civile di Baggiovara, Modena University Hospital, Modena, Italy; Department of Neurology with Experimental Neurology, Charité - Universitätsmedizin Berlin, Corporate member of Freie Universität Berlin and Humboldt Universität zu Berlin, Berlin, Germany; Center for Stroke Research Berlin (CSB), Charité – Universitätsmedizin Berlin, Berlin, Germany; Department of Neurology, Hospital de Egas Moniz, Centro Hospitalar Lisboa Ocidental, Lisbon, Portugal; IRCCS Istituto delle Scienze Neurologiche di Bologna, Department of Neurology and Stroke Center, Maggiore Hospital, Bologna, Italy; Stroke Unit and Department of Neuroradiology, ASST Spedali Civili, Brescia, Italy; Stroke Center EOC, Neurology, Neurocenter of Southern Switzerland, EOC, Lugano, Switzerland; Department of Neurology, Hadassah-Hebrew University Medical Center, Jerusalem, Israel; University of Belgrade, Faculty of Medicine, Neurology Clinic, University Clinical Centre of Serbia, Belgrade, Serbia; Department of Neurology and University Institute of Diagnostic and Interventional Neuroradiology, Inselspital, Bern University Hospital and University of Bern, Bern, Switzerland; Department of Neurology, University Hospital Zurich and University of Zurich, Zurich, Switzerland; Stroke Center, Neurology Service, Lausanne University Hospital and University of Lausanne, Lausanne, Switzerland; Department of Neurology, Amsterdam UMC Location University of Amsterdam, Amsterdam, The Netherlands; Neurology, University of Helsinki and Helsinki University Hospital, Helsinki, Finland; Department of Neurology and Stroke Center, University Teaching and Research Hospital, Kantonsspital St. Gallen, St. Gallen, Switzerland; Neurology - Stroke Unit, Department of Neuroscience, Ospedale Civile di Baggiovara, Modena University Hospital, Modena, Italy; Department of Neurology with Experimental Neurology, Charité - Universitätsmedizin Berlin, Corporate member of Freie Universität Berlin and Humboldt Universität zu Berlin, Berlin, Germany; Center for Stroke Research Berlin (CSB), Charité – Universitätsmedizin Berlin, Berlin, Germany; Department of Neurology, Hospital de Egas Moniz, Centro Hospitalar Lisboa Ocidental, Lisbon, Portugal; IRCCS Istituto delle Scienze Neurologiche di Bologna, Department of Neurology and Stroke Center, Maggiore Hospital, Bologna, Italy; Stroke Unit and Department of Neuroradiology, ASST Spedali Civili, Brescia, Italy; Stroke Center EOC, Neurology, Neurocenter of Southern Switzerland, EOC, Lugano, Switzerland; Department of Neurology, Hadassah-Hebrew University Medical Center, Jerusalem, Israel; University of Belgrade, Faculty of Medicine, Neurology Clinic, University Clinical Centre of Serbia, Belgrade, Serbia; Stroke Center and Department of Neurology, University Hospital Basel and University of Basel, Basel, Switzerland; Department of Neuroradiology, University Hospital Basel, Basel, Switzerland; Department of Neurology and University Institute of Diagnostic and Interventional Neuroradiology, Inselspital, Bern University Hospital and University of Bern, Bern, Switzerland; Department of Neurology and University Institute of Diagnostic and Interventional Neuroradiology, Inselspital, Bern University Hospital and University of Bern, Bern, Switzerland; Department of Neurology and University Institute of Diagnostic and Interventional Neuroradiology, Inselspital, Bern University Hospital and University of Bern, Bern, Switzerland; Stroke Center, Neurology Service, Lausanne University Hospital and University of Lausanne, Lausanne, Switzerland; Department of Neurology, Amsterdam UMC Location University of Amsterdam, Amsterdam, The Netherlands; Neurology, University of Helsinki and Helsinki University Hospital, Helsinki, Finland; Stroke Center and Department of Neurology, University Hospital Basel and University of Basel, Basel, Switzerland; Department of Neurology and Stroke Center, University Teaching and Research Hospital, Kantonsspital St. Gallen, St. Gallen, Switzerland; Neurology - Stroke Unit, Department of Neuroscience, Ospedale Civile di Baggiovara, Modena University Hospital, Modena, Italy; Department of Neurology with Experimental Neurology, Charité - Universitätsmedizin Berlin, Corporate member of Freie Universität Berlin and Humboldt Universität zu Berlin, Berlin, Germany; Center for Stroke Research Berlin (CSB), Charité – Universitätsmedizin Berlin, Berlin, Germany; Department of Neurology, Hospital de Egas Moniz, Centro Hospitalar Lisboa Ocidental, Lisbon, Portugal; IRCCS Istituto delle Scienze Neurologiche di Bologna, Department of Neurology and Stroke Center, Maggiore Hospital, Bologna, Italy; Department of Clinical and Experimental Sciences, Neurology Clinic, University of Brescia, Brescia, Italy; Stroke Program and Department of Medicine and Surgery, University Hospital and University of Parma, Parma, Italy; Department of Neurology, University Hospital Zurich and University of Zurich, Zurich, Switzerland; Department of Neurology and University Institute of Diagnostic and Interventional Neuroradiology, Inselspital, Bern University Hospital and University of Bern, Bern, Switzerland; Stroke Center and Department of Neurology, University Hospital Basel and University of Basel, Basel, Switzerland; Neurology and Neurorehabilitation, University Department of Geriatric Medicine FELIX PLATTER, University of Basel, Basel, Switzerland; Stroke Center and Department of Neurology, University Hospital Basel and University of Basel, Basel, Switzerland; Neurology and Neurorehabilitation, University Department of Geriatric Medicine FELIX PLATTER, University of Basel, Basel, Switzerland

**Keywords:** Endovascular therapy, stroke, age, intracranial hemorrhage, elderly%

## Abstract

**Introduction:**

Data on safety of endovascular therapy (EVT) in the very elderly are scarce. Using data from a large prospective EVT registry, we aimed at providing better evidence for EVT decision-making in patients aged 90 years and older.

**Patients and methods:**

In this multicentre observational study from the EVA-TRISP collaboration outcomes were compared between patients aged ⩾90 years with those aged <90 years using multivariate logistic regression analysis and reporting odds ratios and 95% confidence intervals. Outcomes were occurrence of poor functional outcome in survivors (modified Rankin Scale (mRS) 3–5 if pre-stroke mRS 0–2 and mRS higher than pre-stroke mRS if pre-stroke mRS 3–5), mortality at 3 months after stroke, unsuccessful recanalization (mTICI 0–2a) and symptomatic intracranial hemorrhage (sICH, defined by ECASS-II-/III-criteria).

**Results:**

Of 13,306 eligible patients, 892 were ⩾90 years old (6.7%). The very elderly had a higher median National Institutes of Health Stroke Scale (NIHSS) on admission (16 vs 14) and were more likely to have a pre-stroke mRS of 3–5 (38.0% vs 8.7%). The odds of poor functional outcome (OR_adjusted_ 2.35 (95%-CI 1.87–2.97); 61.6% vs 38.7%), death (OR_adjusted_ 3.04 (95%-CI 2.60–3.55); 53.9% vs 21.3%) and unsuccessful recanalization (OR_adjusted_ 1.34 (95%-CI 1.14–1.57); 32.4% vs 27.2%) were higher in patients aged ⩾90 years. The odds of sICH did not differ (OR_adjusted_ 0.92 (95%-CI 0.66–1.28); 5.1% vs 5.0%).

**Discussion and conclusion:**

EVT-treated stroke patients ⩾90 years had higher odds of poor functional outcome, mortality and unsuccessful recanalization than younger patients. However, the probability of sICH after EVT was not increased. The decision in favor of or against EVT in the very elderly should not be based on age alone.

## Graphical Abstract

**Graphical abstract:**
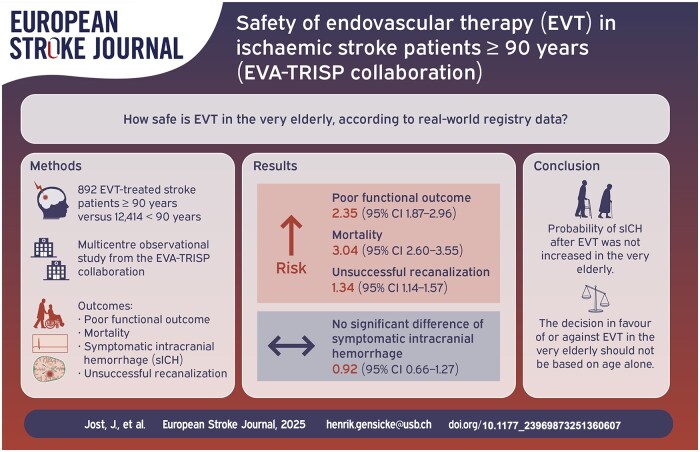

## Introduction

Endovascular therapy (EVT) has changed the clinical outcome of patients with acute ischemic stroke due to large vessel occlusion (LVO). Multiple randomized controlled trials (RCTs)^1–7^ have shown a beneficial effect of EVT on functional outcome (measured by the modified Rankin Scale, mRS) 90 days after stroke onset. However, data from RCTs on EVT-treated patients are limited, with only 91 patients aged ⩾80 years in the RCTs pooled in HERMES^8^ and only 42 patients aged ⩾85 years in a HERMES substudy.^9^ Older age was an exclusion criterion in several RCTs^2,5,7^ probably due to the assumed higher rate of intracranial hemorrhage.^10^ To date, there are no data from RCTs on EVT in nonagenarians. Another important limitation of RCTs on EVT in the elderly is that patients with relevant pre-stroke disability were excluded from all except one RCT (MR CLEAN).^1^ However, pre-stroke disability is frequent in the elderly stroke population (25%–50% of patients).^11^ Data on safety of EVT in nonagenarians consists mainly of observational studies with small sample sizes ranging from 18 to 203 patients ⩾90 years and two meta-analyses. The studies were heterogeneous in regard to the comparison groups and outcomes.^12–25^ Therefore, despite those studies, age remained one of the most relevant factors for withholding EVT in daily routine in a 2019 survey^26^ and there is no consensus on the decision-making process of performing EVT in patients aged ⩾90 years.

The aim of our study was to explore the safety of EVT in patients aged ⩾90 years with an acute ischemic stroke due to a LVO in a large real-world population (using data from the EVA-TRISP collaboration).

## Methods

For this study, we used prospectively collected data from 16 centers of the *EndoVAscular treatment and ThRombolysis for Ischemic Stroke Patients* (EVA-TRISP) collaboration (Supplemental Table S1). EVA-TRISP is an academic multicentre international collaboration with a defined structure, processes and methodology to generate a pooled data registry based on data provided by each participating center.^27^ As done in previous analyses of the database,^28,29^ data for the following variables were used: patient demographics (age, sex, baseline-independency (mRS 0–2),^30^ and baseline mRS), stroke characteristics (National Institutes of Health Stroke Scale (NIHSS) on admission,^31^ territory of stroke (anterior, posterior or both), occluded vessel and/or segment (internal carotid artery, medial cerebral artery M1 or M2, anterior cerebral artery, posterior cerebral artery, basilar artery, vertebral artery or any other occlusion), initial ASPECT score,^32^ time from onset to groin or, if unknown time from last seen well to groin), treatment characteristics (use of IVT in addition to EVT (=bridging therapy), EVT complications, intraarterial thrombolysis therapy (=EVT thrombolysis), mechanical treatment (=stent retriever, aspiration, distal retriever, intracranial balloon angioplasty, permanent intracranial stent alone or in combination), use of general anesthesia, number of passes), systolic blood pressure and glucose levels at admission, medical history^33^ (previous ischemic stroke, coronary artery disease, atrial fibrillation, diabetes mellitus, hypertension, hypercholesterolemia, smoking status (active or stopped less than 2 years ago), prior platelet aggregation inhibition, prior oral anticoagulation) and stroke etiology according to Trial of Org 10,172 in Acute Stroke Treatment (TOAST)^34^ criteria.

Outcomes were poor functional outcome in survivors (up to mRS 5), death (mRS 6) at 3 months after stroke onset, unsuccessful recanalization and symptomatic intracranial hemorrhage (sICH). Poor functional outcome, or the level of disability after stroke, was expressed by the mRS and defined as: a mRS score of 3–5 for patients who had a pre-stroke mRS 0–2 or a mRS higher than the pre-stroke mRS for patients with a pre-stroke mRS 3-5.^35,36^ Post-stroke mRS score was assessed at 3 months via outpatient consultations or telephone calls with patients and/or relatives. As different definitions of poor outcome are common, we also analyzed a simpler but often used alternative definition (poor outcome = mRS 3–6, including deceased patients) as a secondary outcome. sICH was defined, using the European Cooperative Acute Stroke Study (ECASS) II and III criteria, as extravascular intracranial blood on imaging associated with clinical worsening (an increase in NIHSS of more than four points (ECASS II)) or leading to death and was considered to be the cause of clinical deterioration (ECASS III).^37,38^ The criteria used were those provided by the centers, which in most centers were the ECASS II criteria. If both were available, the ECASS II classification was preferred. Follow-up imaging (MRI or CT scan) was performed usually at 24 h after treatment or earlier in case of clinical worsening.^27^ Unsuccessful recanalization of the occluded intracranial vessel was defined by the modified treatment in cerebral infarction (mTICI)^39^ scale 0–2a. Recanalization status was assessed by digital subtraction angiography.

Data were collected from January 2015 to January 2023. Patients with missing data on age or 3-month mRS were excluded.

### Statistical analyses

Statistical analyses were performed with R (Version 4.4.0)^40^ and R Studio (Version 2023.12.1, build 402).^41^ In addition, the following packages were used: “finalifit” and “pheatmap” for missing data pattern analysis,^42,43^ “mice” for the mice-imputation,^44^ “MatchThem” for propensity score matching,^45^ “cobalt” for propensity score diagnostics,^46^ “tidyverse” for data processing.^47^

We investigated the association of age with the outcomes by using age as a categorical variable, comparing patients aged ⩾90 years with those aged <90 years.

### Exploratory data analysis

Continuous data were summarized using the median and interquartile range (IQR) and binary data were summarized as percentages. The Mann-Whitney *U* test was used to compare continuous variables and the χ^2^ test was used for categorical data. Both raw and adjusted *p*-values (Bonferroni’s method) are reported. No formal *p*-value cut-off was defined to assume statistical significance, instead effect sizes and *p*-values are discussed.

### Data cleaning and missing data imputation

The absolute and relative numbers of missing data are reported for all baseline characteristics and outcome variables. We performed limited data cleaning by overwriting logically impossible values and extremely implausible values as missing values (“NA”) as reported in the results section.

To minimize the bias caused by a complete case analysis we performed a multiple imputation by chained equations (MICE) imputation on the dataset containing all variables reported in Table 1. We followed a “multiple imputation, then delete” approach, thus we also used the rows where one or more of the four outcomes were missing for imputation, but we did not use these rows for the analysis of the missing outcomes, that is, the analysis was performed only on reported and not on imputed outcomes. This approach is robust against poor imputation of the outcome and tends to reduce variability in the analysis.^48^ We imputed 100 datasets with 30 iterations per dataset. Numerical data were imputed by predictive mean matching, binary outcomes by logistic regression. All models were calculated on each imputed dataset and the results were pooled according to Rubin’s rules.^49^

**Table 1.** Baseline characteristics.

Variables	All (*n* = 13,306)	Patients <90 years (*n* = 12,414)	Patients ⩾90 years (*n* = 892)	Raw *p*-value	Adj. *p*-value	Missing data
Demographics						
Age, years, median (IQR)	75 (64–82)	73 (63–81)	92 (91–94)	<0.001	<0.001	0 (0%)/0 (0%)
Sex (M)	6863/13,293 (51.6%)	6586/12,402 (53.1%)	277/891 (31.1%)	<0.001	<0.001	12 (0.1%)/1 (0.1%)
Prestroke independent (yes)	10,760/12,049 (89.3%)	10,250/11,226 (91.3%)	510/823 (62%)	<0.001	<0.001	1188 (9.6%)/69 (7.7%)
Prestroke mRS, median (IQR)	0 (0–1)	0 (0–1)	2 (0–3)	<0.001	<0.001	1188 (9.6%)/69 (7.7%)
Stroke characteristics and treatment						
NIHSS admission (0–42), median (IQR)	14 (8–19)	14 (8–19)	16 (11–21)	<0.001	<0.001	318 (2.6%)/19 (2.1%)
Stroke all territories						2476 (19.9%)/189 (21.2%)
Stroke in anterior territory* (yes)	9673/10,641 (90.9%)	9000/9938 (90.6%)	673/703 (95.7%)	<0.001	<0.001	
Stroke in posterior territory* (yes)	1253/10,641 (11.8%)	1207/9938 (12.2%)	46/703 (6.5%)	<0.001	<0.001	
Stroke in both territories (yes)	285/10,641 (2.7%)	269/9938 (2.7%)	16/703 (2.3%)	0.574	1	
ASPECT score, median (IQR)	9 (8–10)	9 (7–10)	9 (8–10)	0.268	1	6811 (54.9%)/462 (51.8%)
Time to groin, median (IQR)	210 (153–312)	210 (153–313)	203.5 (151-301.2)	0.202	1	965 (7.8%)/72 (8.1%)
Bridging (yes)	6258/13,305 (47%)	5847/12,413 (47.1%)	411/892 (46.1%)	0.576	1	1 (0%)/0 (0%)
EVT complications (yes)	1562/9134 (17.1%)	1435/8517 (16.9%)	127/617 (20.6%)	0.02	0.785	3897 (31.4%)/275 (30.8%)
EVT thrombolysis (yes)	688/12,151 (5.7%)	656/11,358 (5.8%)	32/793 (4%)	0.049	1	1056 (8.5%)/99 (11.1%)
EVT mechanical (yes)	11,607/12,594 (92.2%)	10,829/11,747 (92.2%)	778/847 (91.8%)	0.779	1	667 (5.4%)/45 (5%)
EVT general anesthesia (yes)	7190/12,284 (58.5%)	6674/11,452 (58.3%)	516/832 (62%)	0.038	1	962 (7.7%)/60 (6.7%)
EVT number of passes, median (IQR)	1 (1–3)	1 (1–3)	1 (1–2)	0.278	1	9395 (75.7%)/718 (80.5%)
Vital signs and laboratory results at admission						
Admission systolic blood pressure (mmHg), median (IQR)	150 (131–170)	150 (131–170)	159 (138–178)	<0.001	<0.001	765 (6.2%)/50 (5.6%)
Admission glucose (mmol/l), median (IQR)	6.9 (5.9-8.3)	6.9 (5.9-8.3)	7 (5.9-8.4)	0.307	1	902 (7.3%)/59 (6.6%)
Medical history						
Prior ischemic stroke (yes)	1812/12,651 (14.3%)	1707/11,817 (14.4%)	105/834 (12.6%)	0.154	1	597 (4.8%)/58 (6.5%)
Coronary artery disease (yes)	2109/12,047 (17.5%)	1938/11,262 (17.2%)	171/785 (21.8%)	0.001	0.051	1152 (9.3%)/107 (12%)
Atrial fibrillation (yes)	4843/13,283 (36.5%)	4295/12,391 (34.7%)	548/892 (61.4%)	<0.001	<0.001	23 (0.2%)/0 (0%)
Diabetes mellitus (yes)	2492/13,289 (18.8%)	2374/12,397 (19.1%)	118/892 (13.2%)	<0.001	<0.001	17 (0.1%)/0 (0%)
Hypertension (yes)	9068/13,281 (68.3%)	8317/12,390 (67.1%)	751/891 (84.3%)	<0.001	<0.001	24 (0.2%)/1 (0.1%)
Hypercholesterolemia (yes)	6372/13,279 (48%)	6004/12,388 (48.5%)	368/891 (41.3%)	<0.001	0.002	26 (0.2%)/1 (0.1%)
Current smoking (yes)	2426/12,470 (19.4%)	2411/11,597 (20.8%)	15/873 (1.7%)	<0.001	<0.001	817 (6.6%)/19 (2.1%)
Prior antiplatelet therapy	3779/13,046 (29%)	3444/12,165 (28.3%)	335/881 (38%)	<0.001	<0.001	249 (2%)/11 (1.2%)
Prior oral anticoagulation	2455/12,323 (19.9%)	2256/11,467 (19.7%)	199/856 (23.2%)	0.013	0.511	947 (7.6%)/36 (4%)
Stroke etiology according to TOAST criteria						
TOAST all categories						968 (7.8%)/40 (4.5%)
Large vessel atherothromboembolic (yes)	2366/12,340 (19.2%)	2272/11,486 (19.8%)	94/854 (11%)	<0.001	<0.001	
Cardioembolic (yes)	5494/12,340 (44.5%)	4940/11,486 (43%)	554/854 (64.9%)	<0.001	<0.001	
Other (Yes)	850/12,340 (6.9%)	843/11,486 (7.3%)	7/854 (0.8%)	<0.001	<0.001	
More than one (yes)	881/12,340 (7.1%)	827/11,486 (7.2%)	54/854 (6.3%)	0.373	1	
Undetermined (yes)	2707/12,340 (21.9%)	2564/11,486 (22.3%)	143/854 (16.7%)	<0.001	0.007	
Outcomes						
Poor functional outcome at 3 months in survivors (yes)	3673/9266 (39.6%)	3439/8886 (38.7%)	234/380 (61.6%)	<0.001	<0.001	882 (9%)/31 (7.5%)
Death at 3 months (yes)	3127/13,306 (23.5%)	2646/12,414 (21.3%)	481/892 (53.9%)	<0.001	<0.001	0 (0%)/0 (0%)
Symptomatic intracranial hemorrhage (yes)	591/11,790 (5%)	549/10,971 (5%)	42/819 (5.1%)	0.941	1	1443 (11.6%)/73 (8.2%)
Unsuccessful recanalization (yes)	2900/10,495 (27.6%)	2649/9720 (27.2%)	251/775 (32.4%)	0.002	0.941	2694 (21.7%)/117 (13.1%)
Alternative poor functional outcome overall (mRS > 2)	7491/13,306 (56.3%)	6702/12,414 (54%)	789/892 (88.4%)	<0.001	<0.001	0 (0%)/0 (0%)

NIHSS: National Institutes of Health Stroke Scale; sICH: symptomatic intracerebral hemorrhage (according to ECASS II/III criteria).

Poor functional outcome in survivors = modified Rankin Scale 3–5 for patients with a pre-Stroke mRS 0–2 or an mRS 4–5 for those with a pre-Stroke-mRS 3–5. Alternative poor functional outcome overall = mRS 3–6. The Mann-Whitney *U* test was used to compare continuous variables and χ^2^ test was used for categorical data. Adjusted *p*-values are derived from Bonferronis correction applied across the complete table. Missing data are given per group (young vs old) separately.

^*^Including strokes in both anterior and posterior territories.

### Statistical modeling

The association of old age (⩾90 years) with the main outcomes was estimated by calculating odds ratios (OR) with 95% confidence intervals (95% CI), using multivariable binary logistic regression models. Models were adjusted for well-known confounders described in the literature. The models for poor outcome and death at 3 months were adjusted for NIHSS at admission,^50,51^ sex,^52^ EVT complications,^51^ unsuccessful recanalization,^50,51^ prestroke mRS,^51,53^ bridging therapy,^54^ admission glucose,^55^ prior atrial fibrillation,^56^ prior ischemic strokes,^51^ and time to groin^57^ (10 covariates total). The model for sICH was adjusted for sex,^58^ NIHSS at admission,^51,59^ EVT complications,^51^ bridging therapy,^51^ admission systolic blood pressure^59^ and last seen well to groin time^51,58,59^ (six covariates total). The model for incomplete recanalization was adjusted for sex^60,61^, NIHSS at admission,^60,61^ use of general anesthesia during EVT,^62^ bridging therapy,^60,61^ admission systolic blood pressure,^60,61^ prior atrial fibrillation,^60,61^ prior diabetes mellitus^60,61^ and last seen well to groin time^60,61^ (eight covariates total).

The main analysis is the full models using the imputed data set. The following sensitivity analyses were performed: (i) The same models were also calculated using only the complete cases to assess the bias caused by the missing data and their imputation. (ii) Using the imputed data sets, simpler models using only NIHSS on admission and the pre-stroke mRS were calculated for all four outcomes to assess the potential gain from using more complex models. (iii) Using the imputed data sets, inversed propensity score matching was performed to reduce the potential influence of between-group differences in key baseline variables on the outcome analyses. We matched patients according to their propensity to be in the old or young age group. The propensity score was based on the complex models and patients were matched in a 4:1 ratio with nearest-neighbor matching within a caliper of 0.05 standard deviations of the propensity score. We report the unadjusted and adjusted covariate balancing. We then performed unadjusted logistic regression analyses in the matched dataset to assess differences in the four outcomes. (iv) Using the imputed data set and the full models we investigated the alternative definition of poor outcome (mRS 3–6, including patients deceased within 3 months after the stroke).

The following subgroup analysis were performed for all four main outcomes using the imputed data set and the full models: (i) patients with reported ASPECT scores available adding the ASPECT score to the full models as additional independent variable, (ii) only patients aged ⩾90 years and analyzing the effects of prestroke disability (mRS 0–2 vs 3–5), site of infarction (anterior vs posterior, excluding patients with both) and stroke etiology (large artery atherosclerosis vs cardioembolic, excluding patients with any other etiology).

Furthermore, to assess an age-dependent effect, the average marginal effect was calculated for all four outcomes based on the complex models and conditioned on age groups summarized per decade.

### Ethics statement and data sharing policy

The study was approved by the ethics committee in Basel, Switzerland. Each participating center additionally has received the local ethical consent in its country. Datasets generated or analyzed within the present study are available from the corresponding author upon reasonable request. In any such case, data sharing will be subject to individual patient consent processes in the participating centers. The final decision on data sharing will be made by consensus of the EVA-TRISP collaborators.

## Results

**Figure 1.**
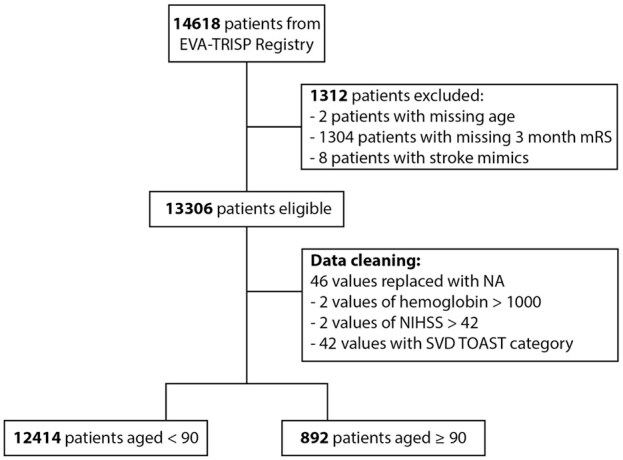
Diagram of patient in- and exclusion and data cleaning. SVD: small vessel disease; TOAST: Trial of Org 10172 in Acute Stroke Treatment.

Out of a total of 14,618 patients, data from 13,306 patients (91.0%) were eligible for analysis (2 (0.01%) excluded due to missing age, 1304 (8.9%) due to missing 3-month mRS, 8 (0.05%) due to being stroke mimics (Figure 1, Supplemental Table S1)). Of those, 892 patients (6.7%) were ⩾90 years of age and 12,414 (93.3%) were <90 years of age. During data cleaning, 46 data points were replaced with missing values (“NA”) due to non-meaningful values (Figure 1).

### Baseline characteristics

In patients aged ⩾90 years, the median age was 92 years (IQR 91–94) and 73 years (IQR 63–81) in the comparison group. Patients aged ⩾90 years were more often women (68.9% vs 46.9%), more often had a pre-stroke disability (38.0% vs 8.7%), presented with a higher NIHSS on admission (median 16 (IQR 11–21) vs 14 (IQR 8–19)) and presented more often with strokes in the anterior (95.7% vs 90.6%) and less often with strokes in the posterior territories (6.5% vs 12.2%; Table 1, Supplemental Table S2A for details on stroke territories). The elderly patients had similar rates of bridging therapy, intraarterial thrombolysis, mechanical treatment, usage of general anesthesia and complications during EVT compared to the control group (Table 1, Supplemental Table S2B for details on EVT complications). The number of passes during EVT was only reported in a limited number of cases (24.3% of young and 19.5% of elderly patients) but there was no evidence of a difference between the study groups (Table 1).

Patients aged ⩾90 years presented with a slightly higher systolic blood pressure (median 159 mmHg (IQR 138–178) vs 150 mmHg (IQR 131–170)) but with similar glucose levels (Table 1).

The rate of patients with prior ischemic stroke(s) was similar in both groups (12.6% ⩾ 90 years vs 14.4%). While patients aged ⩾90 years were more likely to have a history of coronary artery disease (21.8% vs 17.2%), atrial fibrillation (61.4% vs 34.7%) and arterial hypertension (84.3% vs 67.1%), they were less likely to have diabetes mellitus (13.2% vs 19.1%) and hypercholesterolemia (41.3% vs 48.5%) and were decisively less likely to be current smokers (1.7% vs 20.8%). Patients aged ⩾90 years were more likely to receive antiplatelet therapy before the index stroke (38% vs 28.3%) while the rates of oral anticoagulation before stroke were only marginally higher in the elderly (23.2% vs 19.7%). (Table 1).

Furthermore, stroke etiologies differed between the groups with patients aged ⩾90 years more likely to have cardioembolic strokes (64.9% vs 43%) and less likely to have large artery atherosclerosis (11.0% vs 19.8%; Table 1).

Regarding the main outcomes, there was strong evidence that patients aged ⩾90 years had higher frequencies of poor outcome at 3 months in survivors (61.6% vs 38.7%) and death at 3 months (53.9% vs 21.3%). Also, the alternative definition of poor functional outcome (mRS 3–6, including deceased patients) was more often in elderly patients (88.4% vs 54%). There was weak evidence for a higher rate of unsuccessful recanalization in the elderly (32.4% vs 27.2%) while the rate of sICH did not differ between the two groups (5.1% vs 5.0%; Table 1).

The distribution of the mRS at baseline and after 3 months in patients ⩾90 years and <90 years is illustrated in Supplemental Figure S1.

### Missing data and multiple imputation

Over all data given in Table 1, only 10.8% of data was missing (i.e. 89.2% data completeness). This was mainly caused by variables not reported by all centers. The most relevant variables with considerable proportion of missing data were ASPECT score (54.7% missing data) and recanalization status (21.1%). Other variables were territory of infarction (20.0%), EVT complications (31.4%), number of passes (76.00%), coronary artery disease (9.5%) and the TOAST categories (7.6%). Without these variables, only 4.1% of the data was missing (i.e. 95.9% data completeness). The main source of missing data was non-reporting by specific centers, and we therefore assumed that the missing data was missing at random. We calculated the multiple imputations using chained equations with 100 imputations with 30 iterations.

### Multivariable logistic regression models

**Figure 2.**
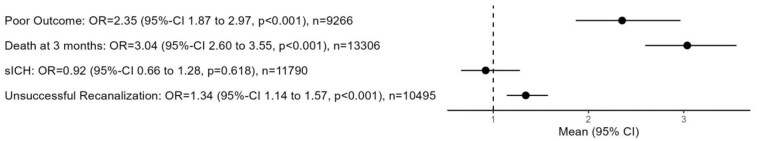
Effect of age group (old vs young) on outcomes. Odds ratios larger than 1 denote larger risk for the patients ⩾90 years. Results are from the full models using the imputed data set. Poor outcome: OR = 2.35 (95%-CI 1.87–2.97, *p* < 0.001), *n* = 9266; death at 3 months: OR = 3.04 (95%-CI 2.60–3.55, *p* < 0.001), *n* = 13,306; sICH: OR = 0.92 (95%-CI 0.66–1.28; *p* = 0.591), *n* = 11,790; unsuccessful recanalization: OR = 1.34 (95%-CI 1.14–1.57, *p* < 0.001), *n* = 10,495.

Using the imputed datasets and the covariates described above we calculated binary logistic regression models. This revealed a larger risk for poor outcome in survivors (OR = 2.35; 95%-CI 1.87–2.97, *p* < 0.001), death (OR = 3.04; 95%-CI 2.60–3.55, *p* < 0.001) and unsuccessful recanalization (OR = 1.34; 95%-CI 1.14–1.57, *p* < 0.001) in patients aged ⩾90 years compared to patients aged <90 years (Figure 2). There was no difference in the rate of sICH (OR = 0.92; 95%-CI 0.66–1.28, *p* = 0.618; Figure 2). The full model results showing all variables are presented in Supplemental Table S3. The strongest binary predictors of poor outcome at 3 months were unsuccessful recanalization (OR = 2.13; 95%-CI 1.9–2.4, *p* < 0.001) and the age group. The strongest binary predictors of death at 3 months were the age group, complications during EVT (OR = 1.73 (95%-CI 1.51–1.99, *p* < 0.001) and unsuccessful recanalization (OR = 1.67; 95%-CI 1.5–1.86, *p* < 0.001). The strongest binary predictors of unsuccessful recanalization were the age group and atrial fibrillation (OR = 0.86 (95%-CI 0.79–0.95, *p* = 0.002). The strongest binary predictor of sICH was complications during EVT (OR = 3.47; 95%-CI 2.84–4.23, *p* < 0.001).

### Sensitivity analyzes

The following sensitivity analyzes for the main outcomes were performed: (i) complete case analysis, (ii) simpler models using only NIHSS on admission and the pre-stroke mRS as covariates and using the imputed datasets, (iii) propensity score matching (as shown in Supplemental Figure S2), and (iv) using an alternative definition of poor outcome (mRS 3–6 vs mRS 0–2). Propensity scores weighted modeling approaches were not possible due to lacking similarity of the groups. We therefore performed a propensity score matching with a ratio of 4:1. A higher ratio did not further increase the number of used cases. Matching was successful and more than 99.0% of the patients aged ⩾90 years could be matched, the remaining 1% of the elderly patients were removed from this analysis. Distribution of the unadjusted and adjusted covariates are given in the supplements (Supplemental Figure S3). All three sensitivity analyses (i–iii) supported the evidence from the main analysis above. The alternative outcome of mRS 3–6 versus mRS 0–2 revealed a higher risk in the elderly compared to the younger patients (OR = 3.90; 95%-CI 3.10–4.91, *p* < 0.001).

### Subgroup analyzes

The ASPECT score was available in 45% of patients (*n* = 6033). Including the ASPECT score as a covariate in the model, the following odds ratios were calculated for poor outcome (OR 2.20, 95%-CI 1.61–3.00; *p* < 0.001), death (OR 3.00, 95%-CI 2.38–3.77; *p* < 0.001), sICH (OR 0.88, 95%-CI 0.55–1.40; *p* = 0.591) and unsuccessful recanalization (OR 1.35, 95%-CI 1.08–1.70; *p* = 0.009).

Among the elderly patients, pre-stroke dependency was associated with poor outcome in survivors but not with death, sICH and unsuccessful recanalization. There was no evidence for a difference in any of the outcomes regarding the stroke territory (anterior vs posterior circulation) or stroke etiology (cardioembolic vs large artery atherosclerosis; Supplemental Table S4).

### Age varying effect on outcomes

**Figure 3.**
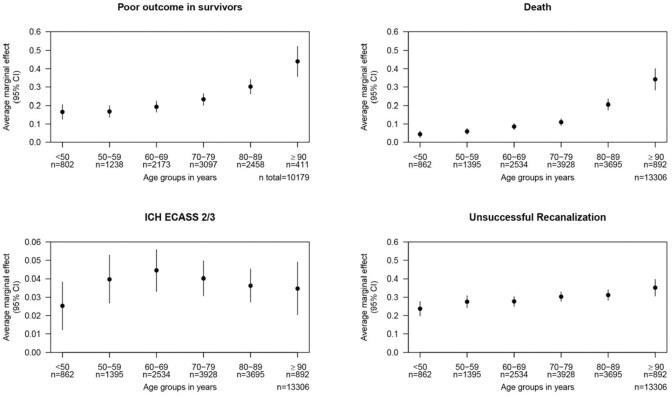
Marginal effects of age groups summarized in decades on the outcomes.

Using the covariates of the full models above and the imputed datasets, we calculated predictive margins. We did not perform a formal test but only report a qualitative assessment of the results, which should be cautiously interpreted due to the larger confidence interval especially in the elderly patients. The risk of poor functional outcome or death increased steadily in patients with increasing age over the decades without apparently reaching a plateau effect. The probability for unsuccessful recanalization also increased with age with a seeming plateau between 60 and 89 years of age, before increasing again for patients aged ⩾90 years. The probability of sICH increased up to age 60 but visually stagnated thereafter (Figure 3).

## Discussion

The main findings of the current study include (1) EVT-treated stroke patients ⩾90 years had an increased probability of poor functional outcome in survivors and death within the first 3 months after stroke as well as a higher risk of unsuccessful recanalization after EVT. (2) The probability of symptomatic intracranial hemorrhage did not differ between patients aged ⩾90 years and aged <90 years.

Evidence from RCTs for benefit of EVT in the elderly is low and especially lacking for those aged ⩾90 years. Furthermore, elderly patients in RCTs likely do not represent those of the real-world because relevant pre-stroke disability had been an exclusion criterion in almost all of the trials.^11^ For patients aged ⩾80 years, a HERMES subgroup analysis^8^ for a mRS distribution shift at 90 days (improvement of 1 or more points) showed a treatment effect favoring EVT in patients aged ⩾80 years. Subgroup analyses for the outcomes mRS score 0–2 at 90 days and mortality also indicated a treatment effect in favor of EVT. However, no analysis was presented for sICH.^8^ A more recent analysis of the HERMES collaboration^9^ focused on outcomes in patients ⩾85 years (77 of 1764 patients). While the probability of poor functional outcome (mRS 5–6: 42.9% vs 74.3%) and mortality (31% vs 54.3%) at 90 days was lower in the intervention group, the frequency of sICH (7.1% vs 5.7%) was similar. Also, when comparing patients aged ⩾85 years to those aged <85 years, no significant differences in the frequency of sICH (7.1% vs 3.6%) and complete recanalization (75% vs 75.5%) were shown, although the absolute (and almost twice as large) difference in the frequency of sICH probably has clinical significance.

While the frequency of sICH in the elderly in the HERMES study was comparable to that in our study, the frequency of poor functional outcome and death was lower in HERMES. This difference is likely explained by a higher proportion of pre-stroke independent patients and younger patients (cut-off at 85 years instead of 90 years in our sample), as well as a different definition of poor functional outcome (mRS 5–6) in HERMES^9^ compared to our study. Although in our study complete recanalization was less frequent in patients aged ⩾90 years compared to the younger group, rates are comparable to those in the elderly of the HERMES study (patients aged >85 years, 75%)^9^ which underlines the plausibility of our data.

When comparing our results with those from other observational studies of the Endovascular Treatment in Ischemic Stroke Registry (ETIS) and German Stroke Registry–Endovascular Treatment (GSR-ET), the frequency among EVT-treated nonagenarians of poor functional outcome (mRS 3–6; 88.4% in EVA-TRISP, 87.3% in ETIS (*n* = 76 nonagenarians)^14^ and 85.8% in GSR-ET (*n* = 203 nonagenarians)^15^), death (53.9% in EVA-TRISP 46.5% in ETIS and 48.9% in GSR-ET), unsuccessful recanalization (32.4% in EVA-TRISP, 28.4% in ETIS and 24.1% in GSR-ET), and sICH (5.1% in EVA-TRISP, 6.8% in ETIS and 3% in GSR-ET) were similar but tendentially worse outcomes in EVA-TRISP. This might be explained by the large proportion of patients with pre-stroke dependency (pre-stroke mRS ⩾3) in EVA-TRISP of 38% and a median pre-stroke mRS of 2. In contrast, patients with pre-stroke dependency were excluded from the ETIS study and less frequent in the GSR-ET study (patients with pre-stroke mRS >3 were excluded and the median pre-stroke mRS was 1).

Furthermore, one meta-analysis, including 657 EVT-treated nonagenarians, found a frequency of poor functional outcome (mRS 3–6) of 78.4%, mortality of 44.4%, unsuccessful recanalization of 19.2% and sICH of 3.5%.^25^ In this study, the rate of poor functional outcome and unsuccessful recanalization was lower than the rates in our study which might be explained by a higher frequency of pre-stroke independency in these studies. However, information on pre-stroke functional status was not provided by the meta-analysis.

Previously, another meta-analysis has compared outcomes of EVT-treated nonagenarians with those of younger stroke patients.^24^ Poor functional outcome (mRS 3–6; 82.6%) and mortality (38.9%) at 90 days were significantly higher in nonagenarians than in younger patients but no significant differences were observed in rates of unsuccessful recanalization and sICH. However, the relevance of this study is limited by the heterogeneity among the included studies and lack on information of the pre-stroke functional status.

Our study included a large population of EVT-treated patients aged ⩾90 years (*n* = 892). This allowed us to better characterize this population, and show differences not only in stroke outcome, but also in pre-stroke functional status, comorbidities and stroke etiology. We found that 38% of EVT-treated patients ⩾90 years were pre-stroke functionally dependent (pre-stroke mRS >2). Furthermore, we found an approximately twofold higher incidence of atrial fibrillation, a corresponding 1.5-fold higher rate of cardioembolic stroke and furthermore, older patients were less likely to suffer from diabetes, hypercholesteremia and were less likely to be active smokers. We hypothesize that this may stem from a double selection bias: (i) The older patients in our database may have benefited from a genetic and/or environmental background to even reach such a high age and (ii) the older patients were obviously deemed fit enough to receive endovascular therapy for reasons other than their age. As our data included only patients who received endovascular therapy, we have no information for which patients it was withheld and why.

The strengths of our study are (1) the large population of EVT-treated patients aged ⩾90 years (*n* = 892) which allows a comprehensive adjustment for confounding parameters; (2) high data completeness (of baseline characteristics); (3) multicentre and multinational design which increases the generalizability of our results; (4) our results were robust when restricted to complete cases, when analyzed with simpler model or with IPSM or in the subgroup with ASPECT score available, which underlines the reliability of our findings.

Our study has the following limitations: (1) The data were collected in European countries and in Israel. Thus, certain risk factors may be different in other regions. (2) We do not have a control group without EVT, thus we cannot estimate the effectiveness of EVT in the very elderly. (3) Compared with RCTs, cohort studies naturally have a higher risk of bias due to the lack of randomization. Therefore, the results of our study should be interpreted with caution. (4) two different scores (ECASS II/III) were used for defining sICH, which may limit the comparability of the results somewhat. Nevertheless, the vast majority was defined by the ECASS II (96.6%) criteria. (5) Despite the overall high data completeness, some important variables (i.e. ASPECTS and status of recanalization) had a considerable proportion of missing data.

### Conclusion

EVT-treated stroke patients ⩾90 years had higher odds of poor functional outcome, mortality and unsuccessful recanalization than younger patients. However, the probability of sICH after EVT was not increased. The decision in favor of or against EVT in the very elderly should not be based on age alone.

## Supplementary Material

sj-docx-1-eso_23969873251360607

## ORCID iDs

Jasmine Jost   https://orcid.org/0009-0009-2964-0814

Lukas Enz   https://orcid.org/0000-0002-6588-2142

Martina B Goeldlin   https://orcid.org/0000-0001-5800-116X

Nabila Wali   https://orcid.org/0009-0000-5410-7805

Nicolas Martinez-Majander   https://orcid.org/0000-0001-8489-7051

Christoph Riegler   https://orcid.org/0000-0002-2478-3500

Matteo Paolucci   https://orcid.org/0000-0002-5347-378X

Hamza Jubran   https://orcid.org/0009-0009-8228-1347

Alexander Salerno   https://orcid.org/0000-0001-8494-5527

Leon A Rinkel   https://orcid.org/0000-0002-0291-8515

Laura Mannismäki   https://orcid.org/0000-0002-3696-7219

Tolga Dittrich   https://orcid.org/0000-0002-9987-3631

Regina von Rennenberg   https://orcid.org/0000-0003-3444-2045

Ronen R Leker   https://orcid.org/0000-0003-4794-0334

Visnja Padjen   https://orcid.org/0000-0002-6126-8305

Urs Fischer   https://orcid.org/0000-0003-0521-4051

Christian H Nolte   https://orcid.org/0000-0001-5577-1775

João Pedro Marto   https://orcid.org/0000-0003-2277-5950

Susanne Wegener   https://orcid.org/0000-0003-4369-7023

Stefan T Engelter   https://orcid.org/0000-0003-3855-6234

## References

[1] Berkhemer OA, Fransen PS, Beumer D. A randomized trial of intraarterial treatment for acute ischemic stroke. N Engl J Med 2015; 372: 394–420. 10.1056/NEJMoa1411587 25517348

[2] Bracard S, Ducrocq X, Mas JL, et al. Mechanical thrombectomy after intravenous alteplase versus alteplase alone after stroke (THRACE): a randomised controlled trial. Lancet Neurol 2016; 15: 1138–1147. 27567239 10.1016/S1474-4422(16)30177-6

[3] Campbell BC, Mitchell PJ, Kleinig TJ, et al. Endovascular therapy for ischemic stroke with perfusion-imaging selection. N Engl J Med 2015; 372: 1009–1018. 25671797 10.1056/NEJMoa1414792

[4] Goyal M, Demchuk AM, Menon BK, et al. Randomized assessment of rapid endovascular treatment of ischemic stroke. N Engl J Med 2015; 372: 1019–1030. 25671798 10.1056/NEJMoa1414905

[5] Jovin TG, Chamorro A, Cobo E, et al. Thrombectomy within 8 hours after symptom onset in ischemic stroke. N Engl J Med 2015; 372: 2296–2306. 25882510 10.1056/NEJMoa1503780

[6] Muir KW, Ford GA, Messow CM, et al. Endovascular therapy for acute ischaemic stroke: the Pragmatic Ischaemic Stroke thrombectomy evaluation (PISTE) randomised, controlled trial. J Neurol Neurosurg Psychiatry 2017; 88: 38–44. 27756804 10.1136/jnnp-2016-314117 PMC5256149

[7] Saver JL, Goyal M, Bonafe A, et al. Stent-retriever thrombectomy after intravenous t-PA vs.t-PA alone in stroke. N Engl J Med 2015; 372: 2285–2295. 25882376 10.1056/NEJMoa1415061

[8] Goyal M, Menon BK, van Zwam WH, et al. Endovascular thrombectomy after large-vessel ischaemic stroke: a meta-analysis of individual patient data from five randomised trials. Lancet 2016; 387: 1723–1731. 26898852 10.1016/S0140-6736(16)00163-X

[9] McDonough RV, Ospel JM, Campbell BCV, et al. Functional outcomes of patients ⩾85 years with acute ischemic stroke following EVT: a HERMES substudy. Stroke 2022; 53: 2220–2226. 35703094 10.1161/STROKEAHA.121.037770

[10] Alawieh A, Starke RM, Chatterjee AR, et al. Outcomes of endovascular thrombectomy in the elderly: a ’real-world’ multicenter study. J Neurointerv Surg 2019; 11: 545–553. 30389899 10.1136/neurintsurg-2018-014289

[11] Creutzfeldt CJ, Levitt MR, Leslie-Mazwi TM. Is endovascular thrombectomy for the very elderly? Stroke 2022; 53: 2227–2229. 35703096 10.1161/STROKEAHA.122.039340 PMC9247017

[12] Friedman I, Naftali J, Pardo K, et al. Efficacy and safety of endovascular treatment in patients older than 90 with acute ischemic stroke: a retrospective cohort study. Front Neurol 2022; 13: 1097423. 36619938 10.3389/fneur.2022.1097423 PMC9815105

[13] Rotschild O, Honig A, Hallevi H, et al. Endovascular thrombectomy is beneficial for functional nonagenarians - a multicenter cohort analysis. J Stroke Cerebrovasc Dis 2022; 31: 106699. 36054973 10.1016/j.jstrokecerebrovasdis.2022.106699

[14] Finitsis S, Epstein J, Richard S, et al. Age and outcome after endovascular treatment in anterior circulation large-vessel occlusion stroke: ETIS Registry results. Cerebrovasc Dis 2021; 50: 68–77. 33321502 10.1159/000512203

[15] Meyer L, Alexandrou M, Flottmann F, et al. Endovascular treatment of very elderly patients aged ⩾90 with acute ischemic stroke. J Am Heart Assoc 2020; 9: e014447. 10.1161/JAHA.119.014447 PMC7335589 32089059

[16] Derraz I, Benali A, Ahmed R, et al. Impact of endovascular reperfusion therapy in nonagenarians with anterior circulation large-vessel ischaemic stroke. Age Ageing 2021; 50: 787–794. 33206940 10.1093/ageing/afaa243

[17] Janssen H, Nannoni S, Francois O, et al. Multicenter, retrospective analysis of endovascular treatment for acute ischemic stroke in nonagenarians. J Stroke Cerebrovasc Dis 2020; 29: 104817. 32689620 10.1016/j.jstrokecerebrovasdis.2020.104817

[18] Rahangdale R, Hackett CT, Cerejo R, et al. Outcomes of endovascular thrombectomy in patients selected by computed tomography perfusion imaging - a matched cohort study comparing nonagenarians to younger patients. J Neurointerv Surg 2022; 14: 747–751. 34475251 10.1136/neurintsurg-2021-017727

[19] Khan MA, Baird GL, Miller D, et al. Endovascular treatment of acute ischemic stroke in nonagenarians compared with younger patients in a multicenter cohort. J Neurointerv Surg 2017; 9: 727–731. 27402857 10.1136/neurintsurg-2016-012427 PMC5226916

[20] Sussman ES, Martin B, Mlynash M, et al. Thrombectomy for acute ischemic stroke in nonagenarians compared with octogenarians. J Neurointerv Surg 2020; 12: 266–270. 31350369 10.1136/neurintsurg-2019-015147

[21] Meyer L, Alexandrou M, Leischner H, et al. Mechanical thrombectomy in nonagenarians with acute ischemic stroke. J Neurointerv Surg 2019; 11: 1091–1094. 31030188 10.1136/neurintsurg-2019-014785

[22] Sweid A, Weinberg JH, Xu V, et al. Mechanical thrombectomy in acute ischemic stroke patients greater than 90 years of age: experience in 26 patients in a large tertiary care center and outcome comparison with younger patients. World Neurosurg 2020; 133: e835–e841. 10.1016/j.wneu.2019.10.024 31614218

[23] Sojka M, Szmygin M, Pyra K, et al. Predictors of outcome after mechanical thrombectomy for acute ischemic stroke in patients aged ⩾90 years. Clin Neurol Neurosurg 2021; 200: 106354. 33172718 10.1016/j.clineuro.2020.106354

[24] Zarrintan A, Musmar B, Ghozy S, et al. Outcomes of mechanical thrombectomy in octogenarians and nonagenarians patients with acute ischemic stroke: a systematic review and network meta-analysis. Eur J Radiol 2024; 176: 111506. 38759542 10.1016/j.ejrad.2024.111506

[25] Bai X, Zhang X, Zhang Y, et al. Mechanical thrombectomy in nonagenarians: a systematic review and meta-analysis. Transl Stroke Res 2021; 12: 394–405. 33532934 10.1007/s12975-021-00894-5

[26] Saposnik G, Menon BK, Kashani N, et al. Factors associated with the decision-making on endovascular thrombectomy for the management of acute ischemic stroke. Stroke 2019; 50: 2441–2447. 31327314 10.1161/STROKEAHA.119.025631

[27] Nordanstig A, Curtze S, Gensicke H, et al. EndoVAscular treatment and ThRombolysis for ischemic stroke patients (EVA-TRISP) registry: basis and methodology of a pan-European prospective ischaemic stroke revascularisation treatment registry. BMJ Open 2021; 11: e042211. 10.1136/bmjopen-2020-042211 PMC8354282 34373287

[28] Filioglo A, Simaan N, Honig A, et al. Outcomes after reperfusion therapies in patients with ACA stroke: a multicenter cohort study from the EVATRISP collaboration. J Neurol Sci 2022; 432: 120081. 34920158 10.1016/j.jns.2021.120081

[29] Traenka C, Lorscheider J, Hametner C, et al. Recanalization therapies for large vessel occlusion due to cervical artery dissection: a cohort study of the EVA-TRISP collaboration. J Stroke 2023; 25: 272–281. 37282374 10.5853/jos.2022.03370 PMC10250869

[30] Broderick JP, Adeoye O, Elm J. Evolution of the modified Rankin Scale and its use in future stroke trials. Stroke 2017; 48: 2007–2012. 28626052 10.1161/STROKEAHA.117.017866 PMC5552200

[31] Lyden P, Brott T, Tilley B, et al. Improved reliability of the NIH stroke scale using video training. NINDS TPA Stroke Study Group. Stroke 1994; 25: 2220–2226. 7974549 10.1161/01.str.25.11.2220

[32] Barber PA, Demchuk AM, Zhang J, et al. Validity and reliability of a quantitative computed tomography score in predicting outcome of hyperacute stroke before thrombolytic therapy. ASPECTS Study Group. Alberta stroke programme early CT score. Lancet. 2000;355:1670-4. DOI: 10.1016/s0140-6736(00)02237-6. Erratum in. *Lancet* 2000; 355: 1670–1674. 10905241

[33] Fluri F, Hatz F, Voss B, et al. Restenosis after carotid endarterectomy: significance of newly acquired risk factors. Eur J Neurol 2010; 17: 493–498. 19922451 10.1111/j.1468-1331.2009.02858.x

[34] Adams HP, Bendixen BH, Kappelle LJ, et al. Classification of subtype of acute ischemic stroke. Definitions for use in a multicenter clinical trial. TOAST. Trial of Org 10172 in acute stroke treatment. Stroke 1993; 24: 35–41. 7678184 10.1161/01.str.24.1.35

[35] Altersberger VL, Rusche N, Martinez-Majander N, et al. Intravenous thrombolysis in patients with ischemic stroke aged ⩾90 years: a cohort study from the TRISP collaboration. Stroke 2022; 53: 3557–3563. 36252105 10.1161/STROKEAHA.122.039426

[36] Gensicke H, Strbian D, Zinkstok SM, et al. Intravenous thrombolysis in patients dependent on the daily help of others before stroke. Stroke 2016; 47: 450–456. 26797662 10.1161/STROKEAHA.115.011674

[37] Hacke W, Kaste M, Bluhmki E, et al. Thrombolysis with alteplase 3 to 4.5 hours after acute ischemic stroke. N Engl J Med 2008; 359: 1317–1329. 18815396 10.1056/NEJMoa0804656

[38] Hacke W, Kaste M, Fieschi C, et al. Randomised double-blind placebo-controlled trial of thrombolytic therapy with intravenous alteplase in acute ischaemic stroke (ECASS II). Lancet 1998; 352: 1245–1251. 9788453 10.1016/s0140-6736(98)08020-9

[39] Zaidat OO, Yoo AJ, Khatri P, et al. Recommendations on angiographic revascularization grading standards for acute ischemic stroke: a consensus statement. Stroke 2013; 44: 2650–2663. 23920012 10.1161/STROKEAHA.113.001972 PMC4160883

[40] R Core Team. R: A Language and Environment for Statistical Computing. Vienna, Austria: R Foundation for Statistical Computing, 2020.

[41] RStudio Team. RStudio integrated development environment for R. Boston, MA: RStudio, PBC, 2020.

[42] Kolde R. pheatmap: Pretty Heatmaps. 2019. https://CRAN.R-project.org/package=pheatmap.

[43] Harrison E, Drake T, Pius R. finalfit: Quickly Create Elegant Regression Results Tables and Plots when Modelling. 2023. https://CRAN.R-project.org/package=finalfit.

[44] van Buuren S and Groothuis-Oudshoorn CGM. mice: multivariate imputation by chained equations in R. J Stat Softw 2011; 45: 1–67.

[45] Pishgar F, Greifer N, Leyrat C, et al. MatchThem: matching and weighting after multiple imputation. Journal 2021; 13: 228.

[46] Greifer N. cobalt: Covariate Balance Tables and Plots. 2024. https://CRAN.R-project.org/package=cobalt.

[47] Wickham H, Averick M, Bryan J, et al. Welcome to the tidyverse. J Open Source Softw 2019; 4: 1686.

[48] von Hippel PT . 4. Regression with missing ys: an improved strategy for analyzing multiply imputed data. Sociol Methodol 2007; 37: 83–117.

[49] Rubin DB. Multiple imputation for nonresponse in surveys. New York: John Wiley and Sons, 1987.

[50] Rogalewski A, Klein N, Friedrich A, et al. Functional long-term outcome following endovascular thrombectomy in patients with acute ischemic stroke. Neurol Res Pract 2024; 6: 2. 38297374 10.1186/s42466-023-00301-4 PMC10832147

[51] Ironside N, Chen CJ, Chalhoub RM, et al. Risk factors and predictors of intracranial hemorrhage after mechanical thrombectomy in acute ischemic stroke: insights from the stroke thrombectomy and Aneurysm Registry (STAR). J Neurointerv Surg 2023; 15: e312–e322. 10.1136/jnis-2022-019513 PMC10962911 36725360

[52] Demel SL, Reeves M, Xu H, et al. Sex differences in endovascular therapy for ischemic stroke: results from the get with the guidelines-stroke registry. Stroke 2022; 53: 3099–3106. 35880521 10.1161/STROKEAHA.122.038491

[53] McDonough RV, Ospel JM, Majoie CBLM, et al. Clinical outcome of patients with mild pre-stroke morbidity following endovascular treatment: a HERMES substudy. J Neurointerv Surg 2023; 15: 214–220. 35210331 10.1136/neurintsurg-2021-018428

[54] Qin B, Wei T, Gao W, et al. Real-world setting comparison of bridging therapy versus direct mechanical thrombectomy for acute ischemic stroke: a meta-analysis. Clinics 2024; 79: 100394. 38820696 10.1016/j.clinsp.2024.100394 PMC11177057

[55] Lau LH, Lew J, Borschmann K, et al. Prevalence of diabetes and its effects on stroke outcomes: a meta-analysis and literature review. J Diabetes Invest 2019; 10: 780–792. 10.1111/jdi.12932 PMC6497593 30220102

[56] Kobeissi H, Ghozy S, Seymour T, et al. Outcomes of patients with atrial fibrillation following thrombectomy for stroke: a systematic review and meta-analysis. JAMA Netw Open 2023; 6: e2249993. 10.1001/jamanetworkopen.2022.49993 PMC9857225 36607633

[57] Fransen PS, Berkhemer OA, Lingsma HF, et al. Time to reperfusion and treatment effect for acute ischemic stroke: a randomized clinical trial. JAMA neurol. 2016;73:190-6. Doi: 10.1001/jamaneurol.2015.3886. Erratum in. *JAMA Neurol* 2016; 73: 481. 26716735

[58] Hao Z, Yang C, Xiang L, et al. Risk factors for intracranial hemorrhage after mechanical thrombectomy: a systematic review and meta-analysis. Expert Rev Neurother 2019; 19: 927–935. 31200607 10.1080/14737175.2019.1632191

[59] Dong S, Yu C, Wu Q, et al. Predictors of symptomatic intracranial hemorrhage after endovascular thrombectomy in acute ischemic stroke: a systematic review and meta-analysis. Cerebrovasc Dis 2023; 52: 363–375. 36423584 10.1159/000527193

[60] Deng G, Xiao J, Yu H, et al. Predictors of futile recanalization after endovascular treatment in acute ischemic stroke: a meta-analysis. J Neurointerv Surg 2022; 14: 881–885. 34544824 10.1136/neurintsurg-2021-017963

[61] Shen H, Killingsworth MC, Bhaskar SMM. Comprehensive meta-analysis of futile recanalization in acute ischemic stroke patients undergoing endovascular thrombectomy: prevalence, factors, and clinical outcomes. Life 2023; 13: 1965. DOI: 10.3390/life13101965 PMC10608522 37895347

[62] Kniep H, Meyer L, Broocks G, et al. Thrombectomy for M2 occlusions: predictors of successful and futile recanalization. Stroke 2023; 54: 2002–2012. 37439204 10.1161/STROKEAHA.123.043285

